# Orbitofrontal cortex functional connectivity changes in patients with binge eating disorder and bulimia nervosa

**DOI:** 10.1371/journal.pone.0279577

**Published:** 2022-12-28

**Authors:** Jaeun Ahn, DeokJong Lee, Jung Eun Lee, Young-Chul Jung

**Affiliations:** 1 Department of Psychiatry, National Health Insurance Service Ilsan Hospital, Goyang, Korea; 2 Institute of Behavioral Science in Medicine, Yonsei University College of Medicine, Seoul, South Korea; 3 Department of Psychiatry, Yongin Severance Hospital, Yonsei University College of Medicine, Yongin, South Korea; 4 Yonsei Empathy Psychiatric Clinic, Seoul, South Korea; 5 Department of Psychiatry, Yonsei University College of Medicine, Seoul, South Korea; 6 Institute for Innovation in Digital Healthcare, Yonsei University, Seoul, South Korea; University of Toronto, CANADA

## Abstract

We aimed to define the shared and unshared functional neurobiological underpinnings of binge eating disorder (BED) and bulimia nervosa (BN). These disorders both involve loss of control over binge eating, but differ based on purging behavior and body image distortion. BED and BN have also been found to show differences in brain activation patterns in reward sensitivity. We enrolled 13 and 12 drug-naive and medication-free women with BED and BN, respectively, and 22 age- and sex-matched healthy controls. We performed an orbitofrontal cortex (OFC)-seeded resting-state whole brain functional connectivity (FC) analysis among the groups. In this study, BED patients exhibited significantly higher impulsivity than controls, whereas the difference in impulsivity between BN and controls was not significant. Participants with BED and BN showed weaker FC between the left lateral OFC and the right precuneus than controls. In the BED only group, the FC strength between these regions was negatively correlated with self-reported impulsivity. In both BED and BN, FC between the left lateral OFC and the right dorsolateral prefrontal cortex was weaker than that in controls. In BED, FC between the left medial OFC and the right cerebellar lobule IV was stronger than that of other groups. Our current results suggest similarities and differences between BED and BN in OFC-seeded FC with respect to reward processing. In particular, FC of the OFC in BED patients showed a significant correlation with their high impulsivity, which may reflect a decline in executive control over binge eating.

## 1. Introduction

Frequent and recurrent binge-eating is a core diagnostic feature shared by binge eating disorder (BED) and bulimia nervosa (BN) [1]. A significant number of patients with these disorders continue their binge eating following treatment, resulting in a poor prognosis and chronic course. Both BED and BN develop in youth and pose high rates of medical and psychiatric comorbidity [1]. A recent epidemiologic study reported that the median onset of BN was 18 years of age, and the median onset of BED was 20 years [2]. In addition to the high disease burden due to onset at a young age, the two diseases share several clinical characteristics. In particular, both diseases are characterized by a loss of executive control over binge eating. Among transdiagnostic dimensional symptom domains, impulsivity has been shown to be related to impairment of executive control [3–5]. Disease models of BN suggest that binge eating begins with emotion-related impulsive behavior [6], whereas patients with BED have high levels of both general impulsiveness and food-specific impulsivity [7, 8]. Therefore, impulsivity is considered a clinical manifestation that is important in both BN and BED, and clarifying neurobiological factors contributing to the pathophysiology of both diseases is necessary.

Although they share several clinical features, BED and BN are distinct diseases that also exhibit some clinical differences. First, BED and BN differ in that BN is accompanied by inappropriate purging behaviors to prevent weight gain. Also, unlike BED, BN involves body image distortion. In addition, researchers have found that BED and BN exhibit different patterns in the main triggers that induce binge eating (e.g., negative affect, dietary lapse) [9]. Additional effort has been made to elucidate the neurobiological pathophysiology underlying these clinical differences between BED and BN. For instance, previous functional magnetic resonance imaging (fMRI) studies using behavioral tasks have suggested that these two diseases differ in reward processing [10]. BED and BN have also been found to show different brain activation patterns in relation to reward processing [11], as well as different reward sensitivity in related brain regions [12]. Since food is a rewarding stimulus, differences in reward processing in the two diseases could be related to the clinical differences in the causative factors of binge eating and behavioral responses to binge eating.

Functional connectivity (FC) analysis, which explores the functional intercorrelation between brain regions, is useful to investigate the operation of functional brain networks. Numerous FC studies have been conducted on eating disorders, including BED and BN. BED and BN commonly show a weakened FC between the frontal regions, which has been suggested to be associated with their diminished executive control over binge eating [13, 14]. Reduced FC in the parietal cortex, which is related to bodily self-consciousness [15], has been observed in both BED and BN [16, 17]. Although these common alterations were found in FC studies on BED and BN, some differences have also been suggested. Most of the preceding FC studies compared BED and BN with healthy subjects, respectively, but there are some studies that have directly compared BED and BN. In one previous study [14], FC of the medial prefrontal cortex (mPFC), the middle frontal gyrus, and the angular gyrus was stronger in BN than BED, and FC of the posterior cingulate cortex (PCC) was stronger in BED than BN. BED and BN also showed significant between-group differences in anterior cingulate cortex (ACC)-seeded FC. The mPFC is suggested to be related to reward learning [18], the ACC to reward prediction error [19], and the PCC to encoding and retrieval of reward values [20]. Taken together, the evidence from preceding FC analysis supports that BED and BN have functional differences in brain regions related to reward processing.

The orbitofrontal cortex (OFC) is one of the major brain regions of the reward network [21]. The OFC is responsible for assessing the value of outcomes and modifying responses accordingly; its dysfunction leads to altered reward processing and an inability to inhibit prepotent responses [22, 23]. In task fMRI studies involving food reward, reactivity of the OFC was similarly increased in BED and BN, compared to healthy subjects [11]. Interestingly, however, there was also a difference in the activity level of OFC between the two [12]. Considering these findings and the importance of OFC in reward processing, FC alterations centered on OFC are suggested in BED and BN. In previous FC studies of BN, FC alterations in cortical-striatal circuits including the OFC have been reported [24–26]. However, no previous studies have explored OFC-centered FC alterations in BED as well as BN.

The present study aimed to identify the shared and unshared functional neural alterations associated with BED and BN. We used OFC seed-based resting-state whole brain FC analysis to investigate the neural network changes involved in these disorders. The OFC consists of subregions that each have a distinct role: the medial OFC encodes the value of rewards, and the lateral OFC is implicated in the inhibitory processes that suppress previously rewarded choices [27, 28]. The lateral and medial OFC form different functional networks and show different FC patterns [29]. Based on previous evidence, we speculated the following concerning OFC-seeded FC of BED and BN: First, FC between the lateral OFC and the inhibitory control-related regions would be weakened in both BED and BN. These FC alterations in the lateral OFC would show a correlation with high impulsivity in BED and BN, reflecting the deterioration of executive control. Second, the FC patterns of medial OFC related to reward sensitivity would be different in BED and BN. As brain regions where the FC differences of medial OFC between BED and BN would appear, regions such as mPFC, ACC, and PCC were predicted. This is based on a previous study comparing FC of BED and BN [14], and this is because these areas are also related to reward processing.

## 2. Materials and methods

### 2.1. Participants

We enrolled 47 psychiatric drug-naïve, medication-free, right-handed women between the ages of 20 and 30 years through an advertisement posted on the Internet. The height and weight of each participant were measured. The presence or absence of psychiatric illness in the subjects were evaluated through the Mini International Neuropsychiatric Interview (MINI) based on the Diagnostic and Statistical Manual of Mental Disorders, fourth edition (DSM-IV) [30]. A psychiatrist confirmed whether the subjects’ diagnosis of eating disorder met the criteria of the DSM-5 through clinical interviews [1]. All participants completed the Korean version of the Eating Attitudes Test-26 (EAT-26) to screen for an eating disorder [31]. Healthy controls (HC) were defined by no history of psychiatric disorder according to the interviewing psychiatrist and an EAT-26 score < 21. Participants with a BMI of < 17.5 kg/m^2^; a history of psychiatric disorder other than eating disorder, use of psychiatric or herbal medications, use of addictive substances other than alcohol or tobacco, traumatic brain injury, neurological illness, or relevant visual defects; or any contraindications to MRI were excluded. Participants were also excluded if they exhibited alcohol abuse or dependence on DSM-IV. Written informed consent was obtained from all subjects after they received a complete description of the study and before they participated in any procedure. This study was approved by the Institutional Review Board of Severance Hospital, Yonsei University.

### 2.2. Procedure

Comorbid psychiatric disorders were evaluated through a structural clinical interview to identify DSM-5 disorders [32]. All participants answered a set of questionnaires, including the Korean version of the Eating Disorder Examination Questionnaire (EDE-Q) [33], the Binge Eating Scale (BES) [34], Beck Depression Inventory (BDI) [35], Beck Anxiety Inventory [36], Barratt Impulsiveness Scale (BIS) [37], and the Revised Questionnaire on Eating and Weight Patterns [38]. Verbal intelligence quotient scores were assessed using the Korean Wechsler Adult Intelligence Scale-IV [39].

After the psychological evaluation, brain MRI was performed. Before the neuroimaging component, participants fasted for 6 hours and then were asked to rate the degree to which they felt hunger on a 7-point Likert scale. Blood glucose tests were conducted to confirm that the participants were in a fasting state.

### 2.3. Image acquisition

Brain MRI was conducted using a 3T Siemens Magnetom MRI scanner (Siemens AG, Erlangen, Germany) equipped with an eight-channel head coil. Whole-brain fMRI data were acquired with a T2-weighted gradient echo-planar pulse sequence (echo time = 30 ms, repetition time = 2200 ms, flip angle = 90°, field of view = 240 mm, matrix = 64 × 64, slice thickness = 4 mm). A 3D structural MRI dataset was obtained for each subject through a T1-weighted spoiled gradient echo sequence (echo time = 2.19 ms, repetition time = 1780 ms, flip angle = 9°, field of view = 256 mm, matrix = 256 × 256, slice thickness = 1 mm). Participants were instructed to stay awake and fixate on a white crosshair at the center of a black screen and avoid engaging in any specific cognitive, lingual, or motor activity. The participants’ motions were minimized in accordance with the best practice for head fixation. The structural image series was inspected for residual motion.

### 2.4. Preprocessing and FC analysis

Imaging data were processed using a Microsoft Windows platform running MATLAB version 9.3 (R2020a; The MathWorks Inc., Natick, MA, USA) and the MATLAB-based CONN-fMRI Functional Connectivity toolbox, version 19.c (Cognitive and Affective Neuroscience Laboratory, Massachusetts Institute of Technology, Cambridge, MA, USA). Visual inspections of images for artifacts were conducted before preprocessing. All images were aligned along the anterior-posterior commissure line, and the anterior commissure of each image was positioned at the origin position. Afterwards, the default CONN preprocessing pipeline was applied. Functional realignment, unwarping, and slice-timing correction were applied. Both functional and structural images were subjected to gray and white matter and cerebrospinal fluid segmentation. Data were spatially normalized in parallel to the Montreal Neurological Institute space. The normalization involves iteratively estimating the posterior tissue probability maps utilizing non-linear spatial transformation from intensity values of the reference image. Functional Images were resliced to a 2-mm isotropic resolution and smoothed with an 8-mm full-width at half-maximum isotropic Gaussian kernel.

After preprocessing, residual movement physiological noise (i.e., respiration, cardiac pulsations, slow involuntary head position motion, or “spike-like” movements) were denoised from the imaging data [40]. Specifically, denoising included temporal despiking, regressing-out confounding factors (i.e., blood-oxygen-level-dependent signal small ramping effects at the beginning of each session, the six rigid body realignment parameters, and their first-order derivatives), applying an anatomical component-based noise correction method (aCompCor, which reduces physiological and movement noise), detrending to remove linear signal drift, and band-pass filtering to restrict the analysis to a range of frequencies of interest (0.008–0.09 Hz).

The ART-based automatic outlier detection was then run for scrubbing: functional volumes were deemed outliers if their signal intensity deviated by more than five standard deviations from the mean signal intensity of the whole series or showed evidence of a displacement of > 0.9 mm relative to the preceding volume. No subjects were removed from the analysis after scrubbing volume censoring because the functional sequences were > 4 min in all cases [40].

Whole-brain seed-to-voxel FC maps for each subject were constructed. The OFC seed regions (left lateral OFC, x = -36, y = 44, z = -10; left medial OFC, x = -17, y = 42, z = -12; right lateral OFC, x = 33, y = 42, z = -9; right medial OFC, x = 11, y = 41, z = -15) were defined as 6-mm radium spheres centered on previously identified coordinates [41]. Correlation coefficients were extracted and converted to z‐values using Fisher r‐to‐z transformation to estimate FC strengths. FC strength estimates were then compared between groups using analysis of variance at each voxel.

### 2.5. Statistical analysis

Two-tailed one-way analyses of variance were used to compare the demographic and clinical characteristics of the participants. Post hoc analysis was Bonferroni corrected. A Pearson correlation analysis tested the associations between FC strength and the BIS scores. All values of FC between OFC seeds and significant brain clusters were entered into the correlation analysis. Statistical analyses were performed with SPSS (version 25; IBM, Armonk, NY, USA), and thresholds for statistical significance were set to *p* < 0.05.

All imaging analyses were corrected for multiple comparisons using a combination of voxel-level thresholds (*p* < 0.001) and cluster extent threshold family-wise error correction (*p* < 0.05). After clusters with significant group differences were evaluated, Bonferroni post-hoc tests were performed to identify the groups that differed from the others.

## 3. Results

### 3.1. Demographic and clinical variables of subjects

A total of 47 female participants (mean age, 23.74 ± 2.2; HC, n = 22; BN, n = 12; BED, n = 13) participated in the study. There was no statistically significant difference in illness duration between the BED and BN groups (Table 1). Both BED and BN groups showed significantly higher EAT-26, EDE-Q, and BES scores than the HC group. BIS score in the BN group was not significantly different with that in the HC group (p = 0.264), while that in the BED group was significantly higher than that in the HC group (p = 0.013). No differences in hunger scale values were reported in the three groups. All participants had blood glucose levels < 110 mg/dL when they participated in the neuroimaging portion.

**Table 1:** Participant demographic and clinical characteristics.

	Group			Statistical results		
	HC (n = 22)	BED (n = 13)	BN (n = 12)	F	P value	Group differences
Age (yr)	23.6±2.3	23.6±2.6	24.3±1.7	0.403	0.671	
Duration of illness (yr)	0	5.0±3.4	7.5±4.0	21.941	<0.001	HC<BED, BN
BMI (kg/m^2^)	21.0±2.3	25.6±3.8	21.5±2.2	12.339	<0.001	HC, BN < BED
EAT-26	5.3±6.1	21.9±12.9	34.3±739	44.276	<0.001	HC<BED<BN
EDE-Q	18.7±11.8	45.4±22.9	55.9±18.6	21.124	<0.001	HC<BED, BN
BES	10.8±5.9	21.8±5.9	26.2±7.3	26.947	<0.001	HC<BED, BN
BDI	6.2±5.3	14.7±8.6	20.4±7.4	17.711	<0.001	HC<BED, BN
BAI	5.6±6.4	12.4±6.6	20.0±9.6	14.959	<0.001	HC<BED<BN
BIS	49.6±7.4	58.0±6.8	54.3±8.5	5.304	0.009	HC<BED
Hunger scale	3.1±1.1	2.6±1.2	3.1±0.8	0.949	0.395	

Notes: Values are expressed as a mean (SD).

Abbreviations: BAI = Beck Anxiety Inventory; BDI = Beck Depression Inventory; BED = binge eating disorder; BES = Binge Eating Scale; BIS = Barratt Impulsiveness Scale; BMI = body mass index; BN = bulimia nervosa, EAT-26 = Eating Attitudes Test-26; EDE-Q = Eating Disorder Examination Questionnaire; HC = healthy controls; IQ = intelligence quotient.

### 3.2. Imaging analysis

**Fig 1:**
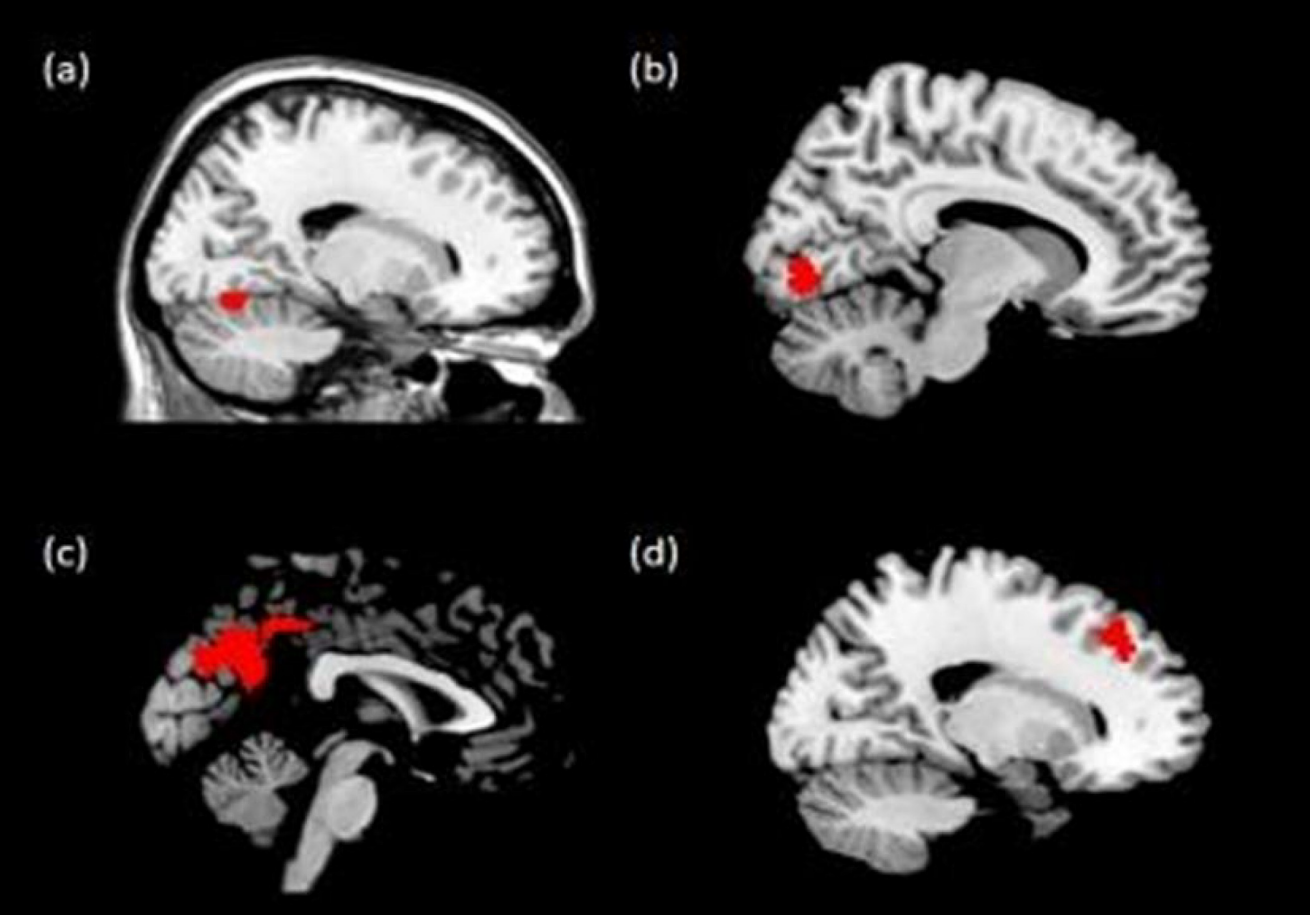
Brain regions whose functional connectivity with the orbitofrontal cortex (OFC) differed between groups: (a) left medial OFC–cerebellar lobule VI, (b) left lateral OFC–right lingual gyrus, (c) left lateral OFC–precuneus, (d) left lateral OFC–right superior frontal gyrus. [voxel-level threshold: uncorrected p<0.001; cluster extent threshold: *p*_FWE_ < 0.05].

The FC analysis results showed weaker FC between the left lateral OFC and right precuneus and right dorsolateral prefrontal cortex (DLPFC) in the BED and BN groups relative to that of the HC group (Table 2, Fig 1). Participants in the BN group had weaker left lateral OFC FC with the right lingual gyrus than participants in the BED and HC groups. Participants in the BED group had stronger left medial OFC FC with the right cerebellar lobule VI than the participants in the BED and HC groups.

**Table 2:** Brain regions with significantly different FC between groups (voxel-level threshold: Uncorrected p<0.001; cluster extent threshold: *p*_FWE_ < 0.05).

Region	Side	k_E_	Z	Coordinates			Post-hoc test
				x	y	z	
**Left lateral OFC**							
Lingual gyrus	Right	135	4.48	10	-78	-4	BED,HC>BN
Precuneus	Right	1906	5.13	12	-54	38	HC>BED,BN
Dorsolateral prefrontal cortex	Right	158	3.44	28	30	46	HC>BED,BN
**Left medial OFC**							
Cerebellar lobule VI	Right	100	4.39	16	-68	-16	BED>HC,BN

Notes: BED = binge-eating disorder; BN = bulimia nervosa; DLPFC = dorsolateral prefrontal cortex; FC = functional connectivity; HC = healthy controls; k_E_ = number if cluster voxels; OFC = orbitofrontal cortex; PFC = prefrontal cortex.

**Fig 2:**
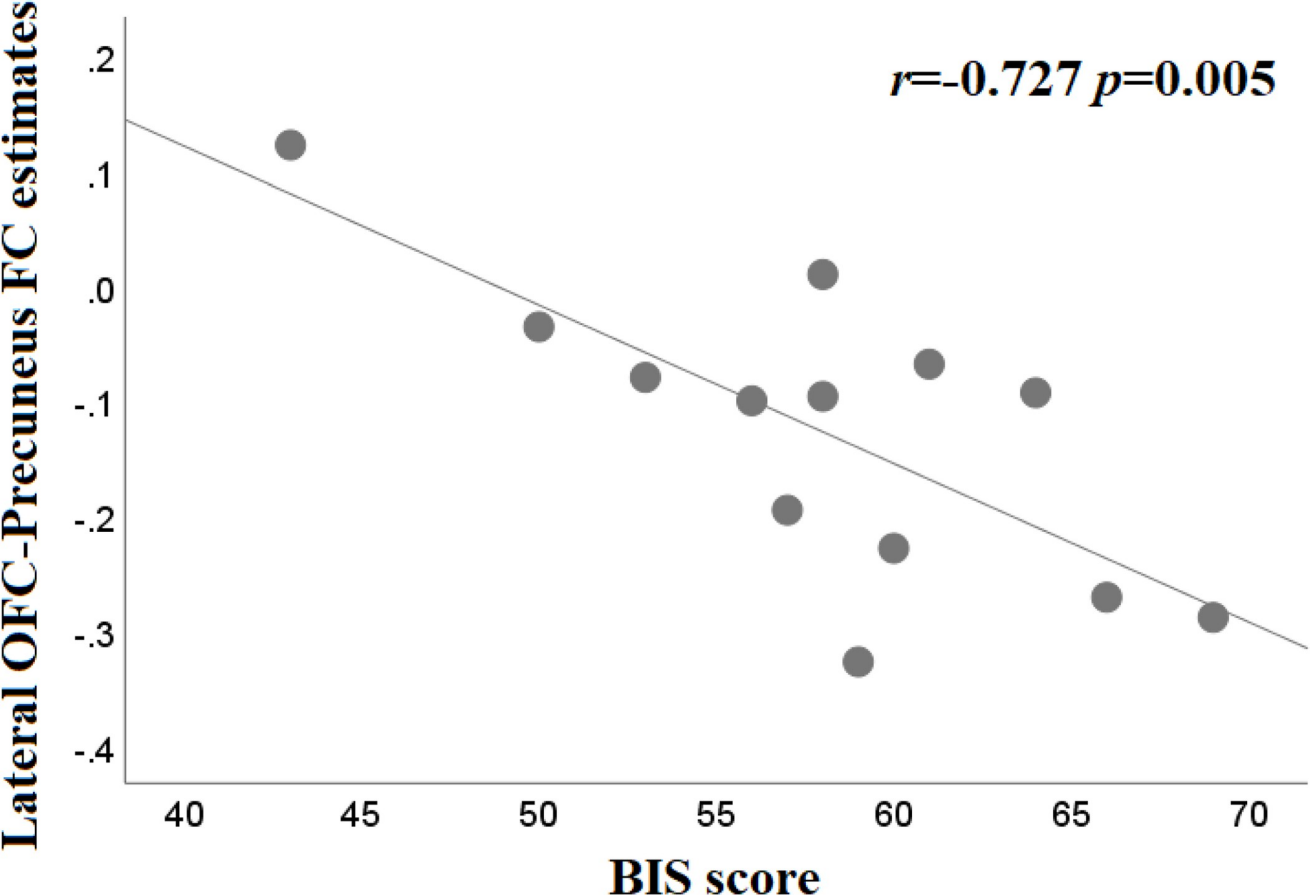
Correlation between Barratt Impulsiveness Scale score and functional connectivity (FC) strength for the lateral orbitofrontal cortex (OFC)-precuneus in participants with binge eating disorder (r = -0.727, p = 0.005).

The stronger FC between the left lateral OFC and the right precuneus was associated with reduced impulsivity as represented by lower BIS scores in the BED group (Fig 2; r = -0.727, *p* = 0.005). The FC between the left lateral OFC and the right precuneus was not correlated with impulsivity in the BN group (r = -0.197, p = 0.540). The other correlation tests showed no statistical significance.

## 4. Discussion

In this study, participants with BED showed significantly higher impulsivity than HCs; however, BIS scores in the BN group were not significantly different from those in other groups. Subjects with BN are generally characterized by high impulsivity, and in a previous study comparing BED and BN, both BN and BED showed high BIS scores [42]. There is a possibility that differences between the BN and HC groups were not significant due to the small sample size. In addition, most of the BN subjects who participated in this study were not obese. One previous study reported that BN subjects with large size of binge eating were more impulsive even within the BN group [43]. Therefore, when interpreting our present findings, we should consider that the BN subjects in this study may not reflect all BN phenotypes.

In the FC analysis, there were OFC-seeded connectivity features that were shared by the disordered groups. We identified weakened FC between the left lateral OFC and the right precuneus and right DLPFC among participants with BED or BN; however, the BED and BN groups also had several distinct findings in OFC-seeded FC. Compared with other groups, the BN group showed decreased lateral OFC FC with the right lingual gyrus, and the BED group showed increased medial OFC FC with the cerebellar lobule IV. These findings suggest that although individuals with BED and BN share recurrent binge eating behaviors, they may differ in underlying neurobiology.

Both BED and BN were associated with decreased FC between the lateral OFC and the right DLPFC, an area that is reportedly involved in the execution of cognitive manipulation [44]. The right DLPFC has been implicated in inhibitory control, and previous research indicates that it may mediate the relationship between motor urgency and response inhibition [45]. Our current findings are consistent with our hypothesis that BED and BN commonly have weak FC between the lateral OFC and inhibitory control-related regions. This is also consistent with previous studies indicating that BED and BN have weakened FC between frontal regions related to cognitive control [10]. In this study, lateral OFC-DLPFC FC did not show a significant correlation with impulsivity in both BED and BN groups. The BIS scale, which evaluates trait impulsivity as a self-report, does not reflect all aspects of inhibitory control equally [46]. In previous research, self-reporting impulsivity did not show associations with some parameters of behavioral paradigms of inhibitory control, suggesting that their relationship may not be a linear relationship [47]. Therefore, investigations of brain-behavior relationships in BED and BN through future studies including behavioral tasks would be needed.

Patients with BED and BN showed decreased FC between the lateral OFC and precuneus. One previous fMRI study reported that weak FC between OFC and precuneus was associated with less goal-oriented and more impulsive tendencies [48]. In the current study, FC between the lateral OFC and precuneus was significantly associated with impulsivity in the BED group. Considering that impulsivity is the most prominent clinical feature of BED, the findings of the current study may have important implications for the identification of the pathophysiology of BED. On the other hand, lateral OFC-precuneus FC did not show a significant correlation with impulsivity in the BN group. This is likely because the number of subjects in this study was small and that the impulsivity of the BN group in this study was not significantly high. Another possibility is that lateral OFC-precuneus FC in the BN group may be related to clinical characteristics other than impulsivity. One previous fMRI study showed that weak FC between the OFC and precuneus was associated with low resilience to psychological stress [49]. Also, higher perceived stress and lower resilience were found to be related to binge eating behavior [50]. This has guided the assumption that individuals with BN are less resilient to psychological stress and more susceptible to stress than HCs are, leading to pathological binge eating behavior. In order to verify this assumption, evaluation of perceived stress and resilience is warranted in future studies.

We found that left medial OFC FC with the cerebellar lobule VI was significantly greater in individuals with BED relative to those in individuals with BN or HC. This was in line with our hypothesis that the difference between BED and BN would appear in medial OFC-seeded FC in relation to reward processing. However, the brain region that showed significant differences between groups in the medial OFC-seeded FC was cerebellar lobule VI, contrary to our expectation (MFC, ACC, and PCC etc.). The cerebellum, which is essential for motor behavior and coordination, has been suggested to be implicated in cognitive function [51]. Previous studies have suggested that the cerebellum is also involved in cognitive control over eating behavior [52]. In particular, cerebellar lobule VI, along with crus I and lobule VIIb, is specifically associated with regulatory control [52–54]. Altered FC between the medial OFC and the cerebellar lobule VI in individuals with BED suggests that they do not adequately recruit brain regions for regulatory control, which may account for their uncontrolled binge eating behavior. Whether FC alterations between the medial OFC and cerebellar lobule VI contribute to BED’s specific pathophysiology should be verified in future studies.

The present study was limited as it only included female participants between the ages of 20 and 30 years. Although women account for higher proportions of BED and BN cases than men [55], our findings may not represent the entire population of individuals with BED or BN. This study was also limited by having recruited relatively small samples of individuals with BED and BN. Studies with larger sample sizes are warranted to validate our findings. In addition, this study did not include the binge eating/purging type of anorexia nervosa (AN). Although this type of AN shows binge eating and purging, it differs from BN in severe calorie restriction, fear of gaining weight, and lack of recognition of underweight. Comparative analysis including binge eating/purging type AN would give a more complete picture of the pathophysiology underlying binge eating behavior in eating disorders. Despite these limitations, the present study benefited from recruited drug-naïve and medication-free participants. Furthermore, given that existing BED and BN neuroimaging studies were mainly performed in European and American populations, our findings expand previous conclusions to Asian populations [6]. In addition, there are few prior studies comparing FC of BED and BN, and in particular, prior FC analysis using OFC as a seed has not been performed to the best of our knowledge.

In summary, the present study investigated brain functional alterations in individuals with BED and BN. We identified OFC-based FC patterns shared by BED and BN: weak FC of the lateral OFC with the precuneus and the DLPFC. On the other hand, there were also some FC differences between BED and BN groups. OFC-seeded FC differences between BED and BN may be related to their different clinical characteristics and pathophysiology. In exploration of brain-behavior relationships, we noted that FC alteration between the lateral OFC and precuneus may contribute to the high impulsivity underlying BED. These findings may help develop future treatment strategies specific to impulsivity in patients with BED.
